# Regulatory Roles of Sortilin and SorLA in Immune-Related Processes

**DOI:** 10.3389/fphar.2018.01507

**Published:** 2019-01-07

**Authors:** Hugo Talbot, Sofiane Saada, Thomas Naves, Paul-François Gallet, Anne-Laure Fauchais, Marie-Odile Jauberteau

**Affiliations:** ^1^Faculty of Medicine, University of Limoges, Limoges, France; ^2^Department of Internal Medicine, University Hospital Limoges Dupuytren Hospital, Limoges, France; ^3^Department of Immunology, University Hospital Limoges Dupuytren Hospital, Limoges, France

**Keywords:** sortilin, SorLA, immune system, cytokines, trafficking, signaling, inflammation, phagocytosis

## Abstract

Sortilin, also known as Neurotensin Receptor-3, and the sorting-related receptor with type-A repeats (SorLA) are both members of the Vps10p domain receptor family. Initially identified in CNS cells, they are expressed in various other cell types where they exert multiple functions. Although mostly studied for its involvement in Alzheimer’s disease, SorLA has recently been shown to be implicated in immune response by regulating IL-6-mediated signaling, as well as driving monocyte migration. Sortilin has been shown to act as a receptor, as a co-receptor and as an intra- and extracellular trafficking regulator. In the last two decades, deregulation of sortilin has been demonstrated to be involved in many human pathophysiologies, including neurodegenerative disorders (Alzheimer and Parkinson diseases), type 2 diabetes and obesity, cancer, and cardiovascular pathologies such as atherosclerosis. Several studies highlighted different functions of sortilin in the immune system, notably in microglia, pro-inflammatory cytokine regulation, phagosome fusion and pathogen clearance. In this review, we will analyze the multiple roles of sortilin and SorLA in the human immune system and how their deregulation may be involved in disease development.

## Introduction

The vacuolar protein sorting 10 protein (Vps10p) domain receptors family is composed of five members: sortilin, SorLA, sorCS1, sorCS2, and sorCS3. In adult human, Vps10p receptors expression was primarily shown in the brain but has also been detected in various other tissues and organs (Hermey, 2009). They also have a dynamic and transient expression during embryonic and post-natal development in rodents, mainly in the nervous system (Hermans-Borgmeyer et al., 1999). Moreover, sortilin is highly expressed in embryonic lung, and SorLA in embryonic lung, kidney, and developing glands (Hermey, 2009). These observations suggest specific functions of sortilin and SorLA in the developing organs.

**FIGURE 1:**
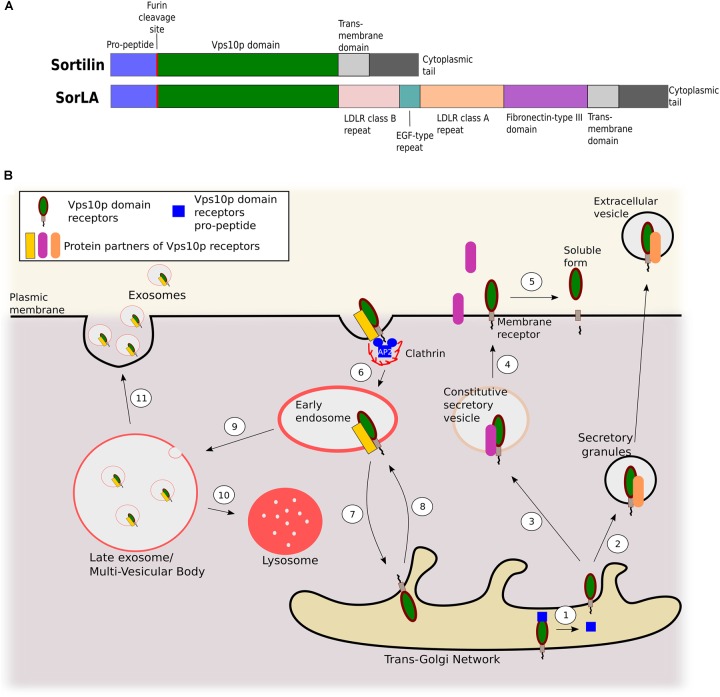
Role of Vps10p domain receptors in intracellular protein trafficking. **(A)** Schematic representation of sortilin and SorLA protein domains. **(B)** Trafficking pathways of Vps10p domain receptors. The propeptide is cleaved by pro-protein convertases in the *trans* Golgi network (step 1). Vps10p domain receptors associated with a protein partner can then be secreted from the TGN to extracellular vesicles through secretory granules (step 2) or addressed to the plasma membrane via constitutive secretory vesicles. Partners can either be anchored to the membrane or secreted as soluble proteins (steps 3–4). Once anchored to the membrane, Vps10p domain receptors can undergo proteolytic cleavage by a disintegrin and metalloprotease (ADAM) 10 or ADAM17, followed by γ-secretase, and be released under a soluble form (step 5). They can mediate internalization of partners from the plasma membrane through AP-2/clathrin-dependent endocytosis (step 6). They can navigate, alone or with partners, between early endosomes and the TGN via the retromer complex (step 7) or anterograde transport via interaction with GCAs/AP-1 (step 8). Early endosomes mature into late endosomes or multi-vesicular bodies (step 9). From there, Vps10p domain receptors and their partners can either be addressed to the lysosome for degradation (step 10) or exocytized in exosomes (step 11).

Since the discovery in the 1990s of sortilin (Petersen et al., 1997; Mazella et al., 1998) and SorLA (Jacobsen et al., 1996), these two proteins have been extensively studied for their functions as regulators of intracellular trafficking through their Vps10p domain. Vps10p is an extracellular ligand binding domain of 700 amino acids folded in a ten-bladed β-propeller (Quistgaard et al., 2009; Kitago et al., 2015). Vps10 domain proteins (Figure 1A) are subjected to various trafficking paths (Figure 1B): (i) transport from the *Trans*-Golgi Network (TGN) to the plasma membrane where they can act as receptors, or be shedded by metalloproteases and γ-secretases to finally be released as soluble proteins (Hermey et al., 2006; Nyborg et al., 2006; Ohwaki et al., 2007; Evans et al., 2011); (ii) clathrin-dependent internalization from the plasma membrane to endosomes (Nielsen et al., 2001; Morinville et al., 2004) and then either to the TGN through the retromer complex (Mari et al., 2007; Seaman, 2007) or to lysosomes for degradation (Dumanis et al., 2015; Tanimoto et al., 2017); (iii) finally they can be exported from the TGN to the extracellular medium through secretory granules (Yang et al., 2011) or extracellular vesicles such as exosomes (Wilson et al., 2014b).

Both sortilin and SorLA have a wide variety of ligands and can either act as sorting regulators or receptors/co-receptors for cell signaling. Therefore, they are involved in many processes depending on cell type, as well as in many associated cellular disorders. Sortilin and SorLA are both involved in neurotensin (NTS) (Mazella et al., 1998; Jacobsen et al., 2001; Mazella, 2001) and neurotrophins signaling (Nykjaer et al., 2004; Teng et al., 2005; Rohe et al., 2009, 2013; Larsen et al., 2010, 2016). They are implicated in Alzheimer’s disease development since sortilin interacts with apoE (Carlo et al., 2013) and SorLA regulates amyloid precursor protein and amyloid-β peptides sorting (Andersen et al., 2005; Offe et al., 2006). Sortilin also regulates glucose transporter (Glut-4) sorting (Pan et al., 2017). Moreover, SorLA has been described as a risk factor for obesity (Whittle et al., 2015; Schmidt et al., 2016). Both proteins are involved in cardiovascular and metabolic diseases, such as atherosclerosis and type-2 diabetes, because of their lipoproteins regulatory functions reviewed in (Schmidt and Willnow, 2016). Finally, sortilin has been shown to be implicated in the development of different cancers (Wilson et al., 2016).

Few studies showed the expression of sortilin and SorLA and their potential functions in lymphoid tissues and bone marrow (BM). Sortilin is expressed in T and B lymphocytes, dendritic cells, NK cells, macrophages and microglia (Martin et al., 2003; Fauchais et al., 2008; Herda et al., 2012; Yabe-Wada et al., 2016) and SorLA expression was detected in monocytes, T and B cells and hematopoietic precursors (Zhang et al., 2000; Sakai et al., 2012). Here, we reviewed their involvement in immune-related processes, their known protein partners during these processes, and the pathologies associated with their deregulation.

## Sortilin, SorLA, and Inflammation

Inflammation is the organism’s response to harmful stimuli. Upon exposure of tissues and organs to pathogens or toxic products, both innate and adaptive immune responses are activated at the inflammatory site. Main inflammation actors are innate and adaptive immune cells (macrophages, monocytes, dendritic cells, T-cells, B-cells…) which are recruited to the inflammatory site via pro-inflammatory chemokines. Inflammation mechanisms are controlled by pro-inflammatory cytokines, inducing complex intracellular signaling pathways. These cytokines are mainly interleukin (IL)-1, IL-6, IL-17, TNF-α and types I, II, and III interferon (IFN). Disruption of these factors may lead to auto-inflammatory and pro-inflammatory disorders (Turner et al., 2014).

### Sortilin, SorLA, and Pro-inflammatory Cytokines

#### Sortilin Regulates Pro-inflammatory Cytokines Exocytosis and Signaling

Recent studies have highlighted the involvement of sortilin in the regulation of cytokines secretion during different immune functions, related to cell cytotoxicity and inflammation, through the control of IFN-γ and IL-6 exocytosis.

These functions were deduced from experimental models of *Sort1* knock-out (sort1^-/-^) C57BL/6 mice. In natural killer cells (NK) and cytotoxic T lymphocytes (CTL), sortilin deficiency impaired the endosomal trafficking of cytolytic vesicles containing granzyme A. An upregulation of vesicle-associated membrane protein 7 (VAMP7), a late endosomal trafficking regulator, was observed due to its diminished lysosomal degradation. This was associated with the increase of granzyme A release and cytotoxic activity of CTL and NK cells (Herda et al., 2012). In this model, IFN-γ retention was detected in the Golgi network of CTLs, NKs, and Th1 cells. Indeed, sortilin interacts with IFN-γ in early endosomes sorting platforms at the TGN–toward different vesicle compartments: recycling endosomes, late endosomes or secretory lysosomes. The consequences of sortilin inactivation are demonstrated both during bacterial infection leading to a decreased IFN-γ release and in experimental autoimmune colitis reducing inflammatory lesions. In these models, TNF-α secretion was not modified and remains independent from sortilin binding and trafficking. Hence, through the regulation of both granzyme A and IFN-γ release, sortilin regulates the immune functions of T lymphocytes and NK cells during adaptive immune responses and target cell killing (Herda et al., 2012).

In addition to INF-γ, sortilin binding to IL-6 was demonstrated in LPS-activated type 1 macrophages (M1). This interaction depends on its extracellular domain and occurs intracellularly to regulate their secretion (Mortensen et al., 2014). IL-6 and IFN-γ effects are synergistic since IL-6 stimulates Th1 activation and IFN-γ secretion, and IFN-γ promotes IL-6 production by macrophages. In a C57BL/6 mouse model of atherosclerosis, the absence of sortilin induced a defect of IL-6 and IFN-γ secretion in activated macrophages and Th1 cells, reducing the inflammatory component of vascular lesions and atherosclerosis, independently of sortilin effect on lipid metabolism. Hence, by directly regulating IFN-γ and IL-6 secretion, sortilin could be a key regulator of inflammatory response increasing inflammatory component of atherosclerosis (Mortensen et al., 2014). Its deregulation in other inflammatory diseases needs to be investigated.

Beside IFN-γ and IL-6, surface plasmon resonance analysis demonstrated that sortilin also binds to other cytokines IFN-α, IL-17A, IL-10, and IL-12 (Yabe-Wada et al., 2016). The functions of sortilin in the exocytic trafficking of IFN-α was demonstrated in plasmacytoid dendritic cells (pDCs), known to secrete IFN-α (Yabe-Wada et al., 2016). This IFN trafficking depends on the dimerization of the sortilin ectodomain in acidic pH conditions encountered in the RE and TGN (Yabe-Wada et al., 2018). Through TLR9 activation, IFN-α release depends on sortilin and is diminished in sortilin-inactivated pDCs, without affecting its RNA transcription. However, a sustained TLR9 activation induces a subsequent negative control of IFN-α release depending on a post transcriptional degradation of sortilin, induced by TLR activation. Given the importance of IFN-α in antiviral immunity or in certain autoimmune diseases such as systemic lupus erythematous, it would be interesting to further study the role of sortilin in the regulation of type I IFN secretion in these related disorders.

**FIGURE 2:**
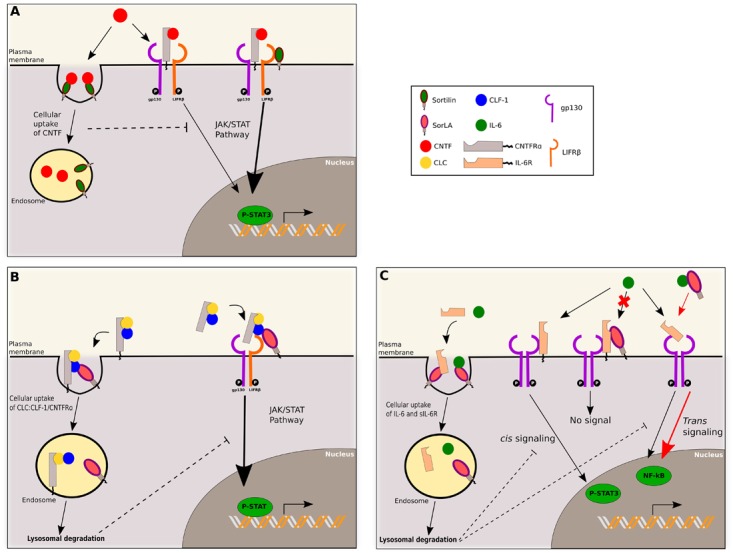
Sortilin and SorLA regulate IL-6 family signaling. **(A)** CNTF binds to CNTFRα, this complex engages the heterodimer gp130/LIFRβ to induce JAK/STAT pathway signaling. Sortilin can bind to LIFRβ and enhance this JAK/STAT signaling. Sortilin also mediates soluble CNTF cellular uptake, and decreases associated signaling. **(B)** SorLA interacts with CLF-1. CLF-1 can form a complex with CLC and CNTFRα. If this complex is soluble, SorLA binds to CLF-1 and concentrates the complex on the membrane, enhancing interaction with gp130/LIFRβ and JAK/STAT signaling. If this complex is anchored to the membrane, SorLA binds to CLF-1 and mediates endocytosis of the complex, followed by its degradation, decreasing associated signaling. **(C)** SorLA can bind to IL-6 or IL-6R. Binding of IL-6 to membrane anchored IL-6R, in association with gp130 homodimer, induces *cis* signaling (JAK/STAT pathway). SorLA can bind IL-6R and inhibits IL-6 binding and subsequent signaling. IL-6 can also engage soluble IL-6R, associated with gp130 homodimer, and induce *trans* signaling (NF-kB pathway). SorLA may bind to soluble IL-6 and act as a stabilizer, enhancing IL-6 half-life, and thus *trans* signaling. Additionally, SorLA negatively controls IL-6 turnover by binding to IL-6 or sIL-6R and mediating their endocytosis, followed by lysosomal degradation.

In addition to the regulation of cytokine exocytosis, sortilin also regulates their signaling as reported for ciliary neurotrophic factor (CNTF) belonging to IL-6 family of cytokines sharing a common gp130 subunit receptor. This family includes IL-6, IL-11, IL-27, leukemia inhibitory factor (LIF), oncostatin M (OSM), ciliary neurotrophic factor (CNTF), cardiotrophin-1 (CT-1), cardiotrophin-like cytokine (CLC), neuropoietin (NP), and IL-31 (Scheller et al., 2011). CNTF activation depends on its binding to the receptor CNTFRα, followed by the recruitment of the heterodimeric complex gp130/LIFRβ receptor, leading to Janus Kinase (JAK) and STAT3 activation/phosphorylation. Sortilin binds the C-terminus tail of CNTF with a high affinity, at a distinct site of CNTFRβ binding, and mediates rapid uptake and clearance of extracellular CNTF. Sortilin also interacts with LIFRβ, and thus facilitates CNTF signaling through the heterodimer gp130/LIFRβ regardless of CNTF or CNTFRα binding to sortilin (Figure 2A). Similar interactions of CLC:CLF-1 with sortilin have been observed. However, its implication in endocytosis and/or secretion process of the cytokine remains unknown. According to the direct interaction of sortilin with LIFRβ subunit, it is worth noting that sortilin facilitates cytokine signaling depending on the LIFRβ subunit receptor. This concerns cytokines CT-1, LIF, OSM that engage the gp130/LIFRβ complex, but not IL-6 which engages the homodimer gp130 (Larsen et al., 2010). Thus sortilin is a key regulator of gp130/LIFRβ-IL-6 family in both physiological and pathophysiological processes, such as B-cell stimulation, the control of regulatory and effector T cells balance, metabolic functions, neural functions or autoimmune diseases (extensively reviewed in Rose-John, 2018). Further studies may highlight an important role of sortilin in the modulation of cell signaling induced by this pleiotropic cytokine family.

#### SorLA Regulates CLF-1 and IL-6 Signaling

Like sortilin, SorLA is implicated into the regulation of IL-6 family signaling as reported in a pro-B lymphocytes murine cell line. Whereas CLC and CLF-1 have a binding site for CNTFRα, only CLF-1 displays a binding site for SorLA. Thus, SorLA interacts either with CLF-1 alone, or with the cytokine CLC:CLF-1 complex via CLF-1 binding as well as with the heterotrimer of CLC:CLF-1 associated with CNTFRα. According to its partners, different functions of SorLA were described (Figure 2B). If the trimer is anchored to the membrane through membranous CNTFRα, SorLA mediates the internalization and lysosomal degradation of the complex and thus downregulates CLC:CLF-1/CNTFRα signaling. On the other hand, if the formed complex includes the soluble form of CNTFRα, SorLA concentrates the complex at the membrane, enhances its interaction with gp130/LIFRβ and thus increases JAK/STAT signaling. So, through interaction with CLF-1 only, SorLA regulates CLC/CNTFRα signaling and turnover (Larsen et al., 2016). SorLA additionally regulates IL-6 signaling (Figure 2C). IL-6 can induce signaling pathways, namely *cis* signaling when it binds to membrane-bound IL-6 Receptor (IL-6R) and *trans* signaling when binding the soluble form of IL-6R (sIL-6R). The complex formed then engages homodimeric gp130 to induce signaling via the JAK/STAT pathway. SorLA binds by its Vps10p domain both IL-6 and IL-6R independently or in complex. When interacting with IL-6 or sIL-6R, SorLA mediates cellular uptake and endocytosis of both proteins. Membrane-bound IL-6R has also been found to interact with SorLA. In such cases, SorLA downregulates *cis* signaling, probably by inhibiting IL-6 binding on IL-6R. The soluble form of SorLA can interact with circulating IL-6, in this complex SorLA acts as a stabilizing carrier protein, increasing IL-6 functional half-life and thus enhancing IL-6 *trans* signaling (Larsen and Petersen, 2017).

Larsen and colleagues highlight in this study a potentially crucial role of SorLA in regulation of IL-6 cytokine family signaling. Once again, the IL-6 family of cytokines is involved in many physiological and pathophysiological processes including, but not limited to, immune response and hepatic acute phase reaction (Rose-John, 2018). Uncovering the role of SorLA in modulation of IL-6 family signaling, could further deepen our understanding of these processes. For example, CLF-1 can interact with p28, a subunit of IL-27, to form a cytokine complex activating NK and T cells in the presence of IL-6Rα (Crabé et al., 2009) and a potential involvement of sortilin or SorLA in this pathway should be investigated given their role in CLF-1 signaling (Larsen et al., 2010, 2016).

#### Inflammatory Conditions Regulate Sortilin Expression

We mentioned previously that sortilin might be a key regulator of pro-inflammatory cytokines. Upon viral infection, viruses are sensed by Toll-like receptors (TLRs) 7 and 9 on pDCs, leading to massive secretion of IFN-α, under the control of sortilin (Yabe-Wada et al., 2016). In the same time, TLR signaling, in response to inflammatory conditions, negatively regulates sortilin expression which impairs IFN-α secretion. Similar findings were observed in adipocytes. Under inflammatory conditions and increase of TNF-α levels, a diminution of both sortilin mRNA and protein were detected (Kaddai et al., 2009; Hivelin et al., 2017), by an unknown mechanism. Recently, several mechanisms are described and correlated with the regulation of sortilin expression, depending on different transcriptional and post transcriptional regulating factors (Sung et al., 2018).

Some mechanisms regulating sortilin expression are linked to transcriptional regulation of *SORT1* gene by different transcriptional factors. In an anti-inflammatory mice model depleted of regulatory T cells (Treg), IFN-γ and TNF-α levels are increased, notably in the liver, in parallel to a local inflammation (Klingenberg et al., 2013). IFN-γ activation decreased *SORT1* transcription and sortilin expression in hepatocytes, through the activation of the JAK/STAT1 pathway and the binding of P-STAT1 to the promoter of sortilin (Pirault et al., 2017).

Another transcriptional regulation of *SORT1* implicates the activating transcription factor 3 (ATF3), a transcriptional repressor and regulator of inflammation pathway. ATF3 is a member of the cyclic adenosine monophosphate (cAMP) response element binding protein (CREB), containing a DNA binding region and a bZIP domain (Chen et al., 1994; Liang et al., 1996; Hai and Hartman, 2001) ATF3 homodimerization represses transcription, but conversely, its heterodimerization with Jun proteins activates transcription (Liang et al., 1996). ATF3 promoter possesses many binding sites of transcription factors such as ATF/CRE (C-rich element), activator protein 1 (AP1) and NF-κB, suggesting that its expression is regulated by stress conditions like pro-inflammatory environment (Hai et al., 1999; Hashimoto et al., 2002). Under stress or pathogenic conditions, TLRs stimulation induces NF-κB activation and ATF3 transcription. ATF3 has been shown to downregulate pro-inflammatory cytokines transcription, notably ofIL-4, IL-5, IL-6, IL-12, IL-13, IFN-γ, IFN-β, TNF-α as well as chemokines CCL1, CCL2, CCL4, CCL5, CCL7, CCL8, and CCL11 (Gilchrist et al., 2006; Khuu et al., 2007; Rosenberger et al., 2008; Hoetzenecker et al., 2012; Lai et al., 2013; Zheng and Abraham, 2013; Boespflug et al., 2014). On the other hand, ATF3 expression is also induced by feedback regulation of some of these cytokines, including IL-6, IFN-γ, and IFN-β (Hai et al., 1999; Ho et al., 2008; Labzin et al., 2015). These results highlight ATF3 role in the modulation of host immune response under stress or physiopathological conditions (Jadhav and Zhang, 2017).

ATF3 binding to the proximal promoter region of *SORT1* represses *SORT1* transcription (Ai et al., 2012). In hepatocytes, endoplasmic reticulum stress induces an increase of ATF3 and thus a reduction of sortilin expression, leading to reduced VLDL clearance and promoting atherosclerosis (Ai et al., 2012; Klingenberg et al., 2013). In parallel, the IFN-γ and TNF-α – induced drop of sortilin expression are both associated with an increase in ATF3 expression.

We hypothesize that regulation of sortilin expression by ATF3 might be of major importance in immunomodulation. Indeed, ATF3 regulates the expression of various pro-inflammatory cytokines and chemokines, and is itself regulated by them. On the other hand, sortilin can modulate the production and secretion of some of those cytokines and is also regulated by them. We believe that a balance between ATF3 and sortilin expression might be of great importance in the modulation of inflammation and immune response. Sortilin promotes inflammation and consequently activates an autoregulation loop by ATF3 activation, although only few studies, all focused on atherosclerosis, confirmed the important role of sortilin/ATF3 axis in innate immunity. These observations should be more deeply investigated especially its potential deregulation in chronic inflammatory diseases.

Moreover, another post transcriptional mechanism controlling sortilin expression was detected under TLR activation. It concerns its mRNA stability depending on CRE, identified in the 3′ UTR of sortilin mRNA. Under physiological conditions, poly-rC binding protein 1 (PCBP1), a protein involved in RNA processing, translation and stability (Makeyev and Liebhaber, 2002), binds to CRE and improves sortilin mRNA stability. TLR signaling activation by viral infection induces an increase in intracellular metal zinc, which in turn leads to PCBP1/CRE dissociation in the 3′ UTR of sortilin mRNA, and finally its degradation. This post transcriptional regulation of sortilin by TLR results in decrease of sortilin protein level and thus activity, and consequently a decrease in pro-inflammatory cytokines production (Yabe-Wada et al., 2016).

### Sortilin, SorLA, and Atherosclerosis

#### Sortilin Regulates Inflammation in Atherosclerosis

Sortilin has been identified as an important regulator of cardiovascular and metabolic disorders. Among them, sortilin is implicated in atherosclerosis and atherosclerotic plaques development through multiple processes, including regulation of calcification (Goettsch et al., 2016), lipoprotein metabolism (Kjolby et al., 2010; Strong et al., 2012), Glut4 biogenesis and glucose uptake in type-2 diabetes (Shi and Kandror, 2005) and arterial wall inflammation (Mortensen et al., 2014; Patel et al., 2015). In this review we will only discuss the relationship between sortilin and the immune system in atherosclerosis (more details on the implication of sortilin in atherosclerosis can be found in recent reviews) (Schmidt and Willnow, 2016; Zhong et al., 2016; Goettsch et al., 2018).

Sortilin is expressed by immune cells, notably macrophages and Th1 cells. In macrophages, sortilin promotes native LDL uptake, leading to the formation of cholesterol-loaded macrophages called foam cells (Patel et al., 2015), implicated in atherosclerosis development (Yu et al., 2013). As described above, sortilin plays an important role in pro-inflammatory cytokines secretion, such as IL-6 in macrophages and IFN-γ in Th1 cells (Mortensen et al., 2014). IL-6 facilitates the secretion of IFN-γ by T cells (Nurieva et al., 2007). Both cytokines have been associated to atherosclerosis promotion (Koga et al., 2007; Schuett et al., 2012). Surprisingly, pro-inflammatory cytokine regulation in macrophages described by Mortensen et al. (2014) in their *sort1^-/-^* mouse model was not confirmed by Patel et al. (2015) despite a reduction in atherosclerosis lesions.

Correlating with previously discussed results on T Reg (FOXP3+)-depleted mice (mimicking hyperinflammation), the increase of IFN-γ and TNF-α downregulates sortilin expression in the liver. This model was marked by extensive atherosclerosis lesions with vascular inflammation. These results are associated with reduced VLDL and chylomicron clearance and augmented plasmatic cholesterol levels, which promote atherosclerosis. They demonstrate that immunity could regulate sortilin-mediated metabolic processes and that chronic inflammation might also promote metabolic disorders and cardiovascular diseases (Klingenberg et al., 2013).

Although very few studies investigated the immunomodulatory role of sortilin in atherosclerosis, we can hypothesize that sortilin promotes formation of foam cells and chronic inflammation in blood vessels inducing atherosclerosis development. This chronic inflammation might in turn downregulate sortilin in the liver and disrupt lipoprotein metabolism, further enhancing atherosclerosis and cardiovascular diseases. Given the small number of studies, some results remain conflicting and need further investigation and clarification.

#### SorLA Modulates Monocyte Migration to Atherosclerotic Lesions

Following vascular injury, circulating monocytes infiltrate the intima and differentiate into macrophages. Those macrophages secrete chemoattractant molecules enhancing inflammatory cells recruitment. One of them, MCP-1 (CCL-2) plays a crucial role in monocyte recruitment and activation. Moreover, an increase of MCP-1 expression has been detected in atheromatous plaques (Lusis, 2000; Charo and Taubman, 2004; McCarthy et al., 2010; Schmidt et al., 2017). Conjugated Linoleic Acids (CLA) were identified as atheroprotective by inhibiting inflammatory cytokines (Yu et al., 2002; Changhua et al., 2005), including MCP-1 secretion and thus monocyte migration, as well as inflammatory phenotypes of activated macrophages (McClelland et al., 2010).

In atherosclerotic tissues, SorLA is overexpressed in monocytes/macrophages, and CLA inhibits its expression through a PPAR-γ-dependent pathway and a decrease of SorLA, reducing migration of monocytes to MCP-1 stimuli. Furthermore, an increase in SorLA was followed by an increase in urokinase-type Plasminogen Activator Receptor (uPAR) known to be involved in the infiltration of intima by monocytes cells and in foam cells formation (May et al., 2002; Gu et al., 2005; McCarthy et al., 2010). Soluble form of SorLA, highly increased in atheromatous plaques, activates uPAR expressed at the cell surface, promoting migration of macrophages and lipids accumulation (Ohwaki et al., 2007).

In atherosclerosis, SorLA exerts a dual role: it is implicated in the regulation of lipolysis (Nilsson et al., 2007, 2008; Klinger et al., 2011; Mendoza-Barberá et al., 2013; Schmidt et al., 2017) and in the regulation of monocyte migration to MCP-1 stimuli. Although these processes were only described in atherosclerosis cases, it would be interesting to study SorLA implication in monocyte/macrophage migration in physiological immune process such as inflammation following antigens stimuli. Hence, uPAR, known as an important player in tissue repair process (Blasi and Carmeliet, 2002), may also implicate SorLA.

### Sortilin, Microglia, and Neuroinflammation

Microglia cells, tissue-based macrophages of the Central Nervous System (CNS), are considered as the key innate immunity cells in the CNS (Gehrmann et al., 1995; Kofler and Wiley, 2011). They have a sentinel immune function, constantly surveying their environment to detect tissue damage and pathogens. As most tissue macrophages, they recognize damage-associated molecular patterns (DAMPs) and pathogen-associated molecular patterns (PAMPs) by high expression of surface receptors such as TLRs, CD68, or CD206, as well as cytoplasmic receptors such as retinoic acid-inducible gene-1-like (RLR) and nuclear oligomerization domain-like receptors (NLR). TLRs signaling results in activation of pro-inflammatory cytokines, such as IL-1β and IL-18, gene transcription and pro-protein synthesis, while RLR or NLR induces formation of an inflammasome (Aravalli et al., 2007; Lehnardt, 2010; Ross, 2010).

Murine and human microglia cells express sortilin but not the two other NTS receptors NTSR1 and NTSR2 (Martin et al., 2003; Patel et al., 2016). Upon stimulation by NTS, sortilin activates both PI3K/AKT and MAPK/ERK1-2 pathways. These signaling pathways induce an increase in pro-inflammatory cytokines and chemokines transcription, including Monocyte Chemoattractant Protein-1 (MCP-1), MIP-2 (or CXCL2), IL-1β, and TNF-α, responsible for inflammatory processes and leucocytes recruitment (Dicou et al., 2004). Upon stimulation by NTS, sortilin induces the migration of murine microglial cells through chemoattractive effects and induces their maturation into pro-inflammatory cytokines producing phagocytes which might contribute to neuroinflammation and development of neurodegenerative disorders (Martin et al., 2003, 2005; Dicou et al., 2004). In human microglia, a similar activation is obtained *in vitro* by NTS, inducing production of pro-inflammatory cytokines IL-1β, CXCL8, CCL2, and CCL5 through PI3K/mTOR activation. These signaling effects were inhibited by mTOR inhibitors such as methoxyluteolin (Patel et al., 2016). A relationship with autism is hypothesized based on the increase in plasmatic NTS levels, evidenced in children with autism spectrum disorders, notably with the accompanying gastrointestinal dysfunction. There is an hypothesized link between such results, neuroinflammation, and the interaction of microglia and microbiome disorders (recently reviewed in Lebovitz et al., 2018), but the mechanisms have yet to be determined.

Sortilin can also regulate brain inflammation by mediating progranulin (PGRN) uptake by microglial cells. PGRN is a growth factor implicated in the regulation of various processes, including wound healing, tumorigenesis and inflammation (recently reviewed in Paushter et al., 2018). PGRN is produced and secreted at high levels by microglia, especially by trauma activated cells, and associated to anti-inflammatory effects by controlling microglial activation, migration, phagocytosis and synapse pruning (Paushter et al., 2018). PGRN could potentially bind to TNFR, inhibiting inflammatory effects by competing with TNF-α (Tang et al., 2011). Sortilin in microglia is able to bind extracellular PGRN and mediate its endocytosis and clearance, thus reducing anti-inflammatory properties (Kumar-Singh, 2011; Chen et al., 2013; Jian et al., 2013). PGRN deficiency causes several neurodegenerative pathologies; PGNR haploinsufficiency is associated with frontotemporal dementia and a total deficiency can cause neuronal ceroid lipofuscinosis. Gene therapies recovering PGRN expression have beneficial effects on the patients affected with frontotemporal dementia or neuronal ceroid lipofuscinosis, and a combined inhibition of sortilin in microglia could reduce PGRN clearance, increasing extracellular PGRN concentration and potentially enhancing its beneficial effects (Arrant et al., 2018).

As microglia are activated via sortilin, its involvement in the development of Multiple Sclerosis has been studied through a model of experimental-induced encephalomyelitis in *sort1^-^*^/^*^-^* mice. Whereas sortilin is expressed in mouse microglia and dendritic cells, its loss reduced the antigen-processing ability in dendritic cells but did not affect the development and progression of brain lesions, suggesting that sortilin did not control this autoimmune encephalitis (Reuter et al., 2015). This discrepancy with the models previously described may be related to the acute experimental model and that sortilin regulatory functions are more relevant in chronic inflammatory models.

### Sortilin, Inflammation, and Cancer?

To further expand on the implication of sortilin in inflammatory context, we open perspectives on cancer-mediated inflammation. As previously discussed, while sortilin deregulation were well characterized in neurodegenerative and cardiovascular diseases (Wilson et al., 2014a; Schmidt and Willnow, 2016), the involvement of sortilin in cancer cells homeostasis remains misunderstood and subject to controversies. Indeed, sortilin participates actively in the release of growth factors promoting thereby autocrine survival loops through “sustained proliferative signaling,” a central hallmark of cancer. Inversely, a cell suicide may triggers following the association of the sortilin with the death domain receptor p75, alimenting thus controversies toward sortilin. Surprisingly, while sortilin regulates the immune functions of T lymphocytes and NK cells, no data are stated whether sort1^-/-^ mice develop spontaneous tumors. Likewise, no clinical data stated about the expression of sortilin in tumor-infiltrating immune cells. Intriguingly, while immune surveillance remains a crucial barrier against transformed cells, immune cells play an important role in tumor initiation, progression, and invasion. Indeed, pre-neoplastic lesions trigger pro-inflammatory cells recruitment and infiltration. For example, gastric cancer cells express both IL-6 and its receptor and may illustrate an indirect role of sortilin mediated pre-metastasis niche formation. Indeed, infiltrated-inflammatory cells release IL-6 upon the dependence of sortilin. Exogenous IL-6 promotes the proliferation and the invasion of gastric cells as well as the secretion of the vascular endothelial growth factor C (VEGFR-C) promoting lymphatic drainage. Thus, the release of IL-6 toward neoplastic lesion promotes lymphangiogenesis, a first step before lymph node invasion (Zhao et al., 2016). Interestingly, sortilin expression is correlated with breast cancer aggressiveness and lymph node metastasis (Roselli et al., 2015). Interestingly, sortilin is also involved in the release of progranulin by breast cancer cells, inducing migration and cancer stem cell expansion (Rhost et al., 2018). However, no data stated about the expression of sortilin in infiltrated-inflammatory cells as well as the implication of sortilin in the release of IL-6 from cancer cells.

In conjunction with IL-6, IFNγ plays a major role in modulation of the cancer immune network to inhibit or promote tumor progression (Landskron et al., 2014; Shalapour and Karin, 2015; Mojic et al., 2017). Through regulation of IFN-γ and IL-6 cytokine family, we speculate that sortilin could be involved in tumor microenvironment inflammation and promote tumor progression.

## Sortilin and Phagocytosis/Scavenging

### Sortilin Mediates Macrophage Antigen Scavenging

**FIGURE 3:**
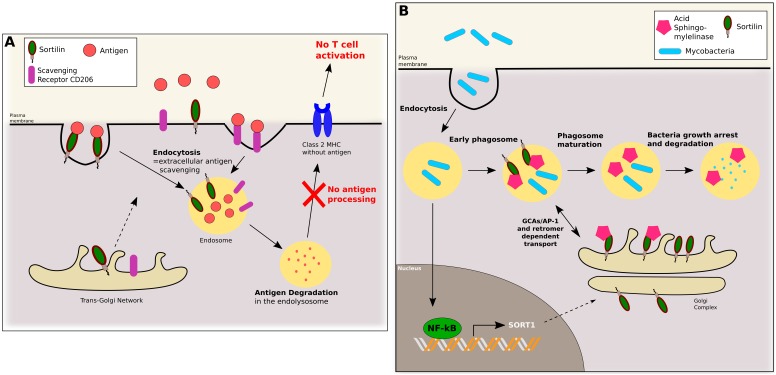
In macrophages, sortilin is implicated in antigen scavenging and phagosome maturation. **(A)** Sortilin and scavenger receptor CD206 are addressed from the TGN to the plasma membrane. In Alternatively Associated Macrophages (AAMs), sortilin can bind extracellular antigens and mediate their endocytosis. Antigens are then degraded in the lysosome, and AAMs being incapable of antigen processing, antigen peptides are not presented to T cells by class 2 MHC. **(B)** Upon phagocytosis of mycobacteria by macrophages, NF-kB pathway is activated, inducing *SORT1* transcription. Sortilin is then transported and vehicles Acid Sphingomyelinase, to early phagosomes. This step is crucial to phagosome maturation and mycobacteria growth arrest and degradation.

Alternatively activated macrophages (AAM), M2-type macrophages, are obtained by stimulation of macrophages by Th2 lymphocytes producing anti-inflammatory cytokines such as TGF-β, IL-4, IL-10, IL-13. These macrophages are characterized by an increase of innate immunity markers, class II MHC and CD14 expression, an increased phagocytic activity and a decreased antigen processing ability (Tzachanis et al., 2002; Gordon, 2003; Martinez et al., 2008; Mosser and Edwards, 2008). AAM showed augmented levels of known scavenger receptors Macrophage Mannose Receptor (or CD206) and sortilin expression. CD206 and sortilin might participate to the increased extracellular antigen scavenging. Associated with AAM inability to process scavenged antigens, this process participates in T cell response inhibition, which is essential for transplantation tolerance (Tzachanis et al., 2002). By regulating antigen clearance by macrophages, sortilin might be indirectly implicated in modulation of T cell mediated immunity and transplant rejection (Figure 3A).

### Sortilin Regulates Phagosome Maturation and Lysosomal Enzyme Delivery

Fusion between the phagosome and the lysosome is a key event in mycobacteria elimination by macrophages. It allows phagolysosome formation, able to efficiently eradicate the infection. Upon infection by the non-pathogenic mycobacteria *Mycobacterium smegmatis*, macrophages activate the NF-κB pathway, inducing phagolysosome fusion and mycobacteria killing. NF-κB activation results in a *de novo* synthesis of both lysosomal enzymatic components and proteins involved in intracellular trafficking, including sortilin (Gutierrez et al., 2008). In macrophages, sortilin is located toward the Golgi apparatus and acquired by phagosomes after their internalization. Sortilin interacts with two lysosomal proteins, Acid Sphingomyelinase (ASM) and Prosaposin (PS), and is required for their delivery from the Golgi to the intracellular phagosomes containing bacteria (Wahe et al., 2010). Likewise, sortilin is required for the targeting of the lysosomal proteases cathepsin D and H (Canuel et al., 2008). Sortilin and ASM are acquired in phagosomes at the early stages of mycobacteria infection. Sortilin and phagosome interaction (and subsequent delivery of ASM) is dependent of both anterograde transport and the retromer complex. Anterograde transport is dependent on interaction of sortilin with GGA/AP-1 complex through the dileucine motif 829LL in the sortilin cytoplasmic tail, and retromer complex interaction is dependent on sortilin residues 787 to 792 (FLVHRY) and cysteine 783 palmitoylation, also in the sortilin cytoplasmic tail. Upon infection by *Mycobacterium bovis* or *tuberculosis*, lack of sortilin elicits an increase of bacteria replication in macrophages, increasing neutrophils lung infiltration and pathogenesis (Vázquez et al., 2016).

In conclusion, macrophages infection by mycobacteria triggers the NF-κB pathway stimulating sortilin and ASM *de novo* synthesis. Then, sortilin transports ASM from the TGN to phagosomes through interaction of its cytoplasmic tail with GGA/AP-1 and the retromer complex essential for anterograde and retrograde transport. Hence, in concert, both sortilin and ASM delivery are required for phagosome maturation, bacteria growth restriction and efficient elimination (Figure 3B).

## SorLA May Be Implicated in Maintaining the Hematological Pool

Hematopoietic Stem and Progenitor Cells (HSPCs) differentiation from immature quiescent self-renewable cells into mature and proliferating cells, is planned by crucial events such as their adhesion to bone marrow cells. The BM pool of HSPCs is regulated by modulation of various molecules under hypoxic conditions (Chow et al., 2001; Lévesque et al., 2010; Trumpp et al., 2010; Suda et al., 2011). Among them, uPAR has been identified as a regulator of HSPCs adhesion in osteoblastic niches as well as a regulator of HSPCs proliferation and marrow pool size (Tjwa et al., 2009).

High levels of SorLA have been observed in immature hematopoietic precursors both at the plasma membrane and in soluble form following proteolytic shedding (Zhang et al., 2000; Hermey et al., 2006). Under hypoxic conditions, intracellular and soluble SorLA levels are increased after HIF-1α binding on *SorLA* promoter. Both soluble SorLA and uPAR are upregulated by hypoxia in the BM niche. Soluble SorLA interacts with membrane bound uPAR in immature hematological cells and modulates its activity, enhancing HSPCs adhesion to BM stromal cells, maintaining thus a normal hematological cell pool size (Nishii et al., 2013).

## Conclusion and Remarks

**FIGURE 4:**
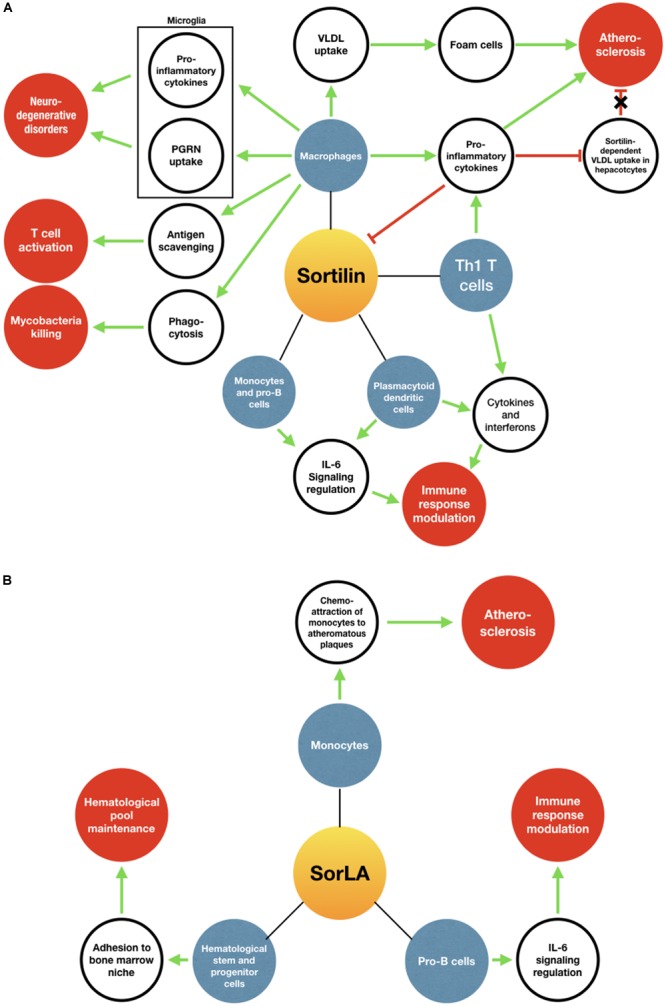
Implications of sortilin **(A)** and SorLA **(B)** in immune-related processes. Blue circles represent cellular models expressing sortilin or SorLA. Blank circles represent pathways/processes/proteins modified by sortilin or SorLA. Red circles represent physiological or pathological processes induced. Green arrows mean a stimulating role, while red arrows mean an inhibition. The red arrow with a black cross means that the inhibition is lifted.

Sortilin and SorLA play incredibly versatile functions depending on their “interactome.” Studies to date emphasize their roles in NTS and neurotrophin signaling pathways in cancer progression, and in cardiovascular, metabolic and neurodegenerative disease. In the present review, we highlighted some of their roles in the immune homeostasis (summarized in Figure 4). Sortilin regulates the production and exocytosis of pro-inflammatory cytokines and is in turn downregulated by them. Both sortilin and SorLA are implicated in the modulation of IL-6 family cytokines signaling and turnover, which could potentially be of major significance in IL-6-dependent immune processes. Sortilin also stimulates neuroinflammation through both pro-inflammatory cytokines release and PGRN clearance by microglia. Furthermore, ATF3 identified as a sortilin expression repressor, may thus have a key immunomodulatory effect by inhibiting inflammation. However, sortilin and SorLA are not only involved in inflammation. Indeed, sortilin is expressed by macrophages and is implicated in antigen clearance, probably impacting T cell immunity and graft tolerance, and is also required during the maturation stages of phagolysosomes formation in cases of infection by mycobacteria. Interestingly, SorLA seems to be important for the differentiation of hematological stem cells in the BM and the maintenance of a normal hematological pool size.

Taken together these observations suggest that sortilin and SorLA may be involved in innate immunity, through regulation of inflammation and phagocytosis, as well as in adaptive immunity by controlling immune cells maturation and modulating T and NK cells activation. For now, only few studies high lighted a role for sortilin and SorLA in normal and/or pathological immunity, however, deeper investigations might identify unknown news functions in immunomodulatory pathways.

## Author Contributions

HT wrote the original draft of the manuscript and created the figures. SS, TN, P-FG, A-LF, and M-OJ reviewed and edited the manuscript and supervised this work.

## Conflict of Interest Statement

The authors declare that the research was conducted in the absence of any commercial or financial relationships that could be construed as a potential conflict of interest.
